# Sensing-range-tunable pressure sensors realized by self-patterned-spacer design and vertical CNT arrays embedded in PDMS

**DOI:** 10.1039/d0ra06481e

**Published:** 2020-09-10

**Authors:** Chao Xie, Min Zhang, Wei Du, Changjian Zhou, Ying Xiao, Shuo Zhang, Mansun Chan

**Affiliations:** School of Electronic and Computer Engineering, Shenzhen Graduate School, Peking University Shenzhen 518055 China zhangm@ece.pku.edu.cn; School of Electronics and Information, South China University of Technology Guangzhou 510641 China; Department of Electronics and Computer Engineering, Hong Kong University of Science and Technology Hong Kong

## Abstract

A pressure sensor design suitable for a broad sensing range with high sensitivity and good stability is highly desirable for the detection of various pressures and meeting the requirements of different applications. Herein, we report sensing-range-tunable piezoresistive pressure sensors realized by self-patterned-spacer design. In the sensors, the two CNT-array layers embedded in PDMS are separated by the proposed self-patterned spacer. With this structure, the realized sensors with large initial resistance designed show tunable response thresholds from 300 Pa to 6.5 kPa while maintaining high sensitivity, which are realized by controlling the spacer thickness and the CNT length. Besides, the vertical CNT arrays have a large specific surface area, which can dramatically change the resistance of the pressure sensors and lead to high sensitivity with nearly 50 kPa^−1^. Benefiting from the designs of the self-patterned spacer and the advantageous combination of CNTs and PDMS, the pressure sensors also exhibit a rapid response/relaxation time of 24/32 ms, and good long-term stability with durability test over 10 000 loading/unloading cycles. On the other hand, the realized pressure sensors with small initial resistance designed show a typical piezoresistive characteristic. For applications, the pressure sensors with large initial resistance designed are suitable for the anti-noise applications with pressure thresholds to filter unnecessary noise and save power consumption, while the pressure sensors with small initial resistance designed show the capability of detecting mechanical forces and monitoring human physiological signals. Moreover, the self-patterned design and fabrication method of the spacers also show potentials to be applied in the existing works to further enhance or adjust the performance of those pressure sensors, showing great flexibility. This design demonstrates great potentials to be applied in future advanced flexible wearable systems such as health monitoring, human–machine interaction and the Internet of Things.

## Abstract 2

A pressure sensor design suitable for a broad sensing range with high sensitivity and good stability is highly desirable for the detection of various pressures and meeting the requirements of different applications.

## Graphical abstract

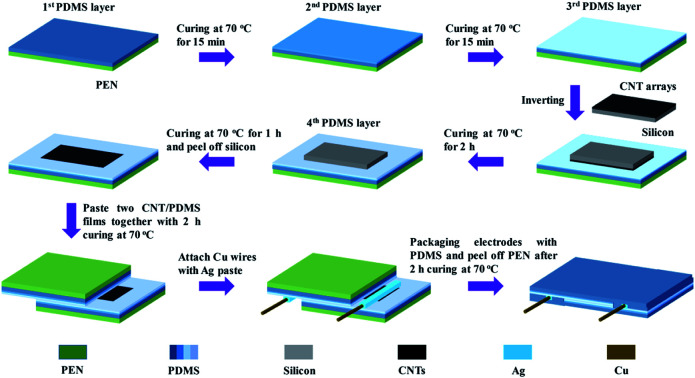

## Introduction

Skin is a vital organ of human body that can protect humans from harm. By mimicking the sensing capabilities of human skin, scientists have developed electronic skin (e-skin) that can sense factors such as pressure,^1–3^ strain,^4–6^ temperature,^7,8^ and humidity,^9,10^ showing great potentials in health monitoring, robotics, and the Internet of Things (IoTs).^11–15^ As the integral part of e-skin, the pressure sensor can convert the sensed external pressure into electrical signals, which plays an important role in disease diagnosis and motion recognition.^16,17^ Pressure sensors can work with different devices such as resistors,^1–3^ capacitors,^18–20^ piezoelectric,^21,22^ and transistors.^23^ Among them, the piezoresistive pressure sensor has been widely studied for its high sensitivity and low detection limit.^1–3^

To meet the various sensing requirements, pressure sensors are also required to be sensitive in various ranges, particularly in high-pressure ranges. Scientists have taken measures to broaden the sensing range of pressure sensors to realize this goal in recent years. Most of them show a higher sensitivity (11.28–57 kPa^−1^) in the initial stage and a lower sensitivity (0.33–1.08 kPa^−1^) in the high-pressure ranges.^3,24,25^ For ultrawide pressure ranges, X. Li *et al.* introduced a flexible piezoresistive pressure sensor based on the polyurethane sponge coated with MXene sheets, showing sensitivities of 0.014 kPa^−1^, 0.015 kPa^−1^ and 0.001 kPa^−1^ in the ranges of 0–6.5 kPa, 6.5–85.1 kPa and 85.1–237.5 kPa, respectively.^26^ When the working range is extended to the order of megapascal, S. Doshi and E. Thostenson have reported a flexible carbon nanotube-based pressure sensor for a ultrawide sensing range from 0.0025 to 40 MPa, while the sensitivity degenerated to 0.05 MPa^−1^.^27^ In general, their sensitivity will decrease with the increase in pressure, which is determined by the characteristics of piezoresistive pressure sensors themselves. In other words, the high sensitivity and broad working range are contradictory to each other. Moreover, in some applications, it is necessary for the pressure sensors to respond sensitively only after exceeding a certain pressure, instead of responding constantly in a broad range, such as a flexible keyboard with a certain threshold to avoid mis-touch and flexible data acquisition systems, which need to avoid impact from unwanted noises and save power consumption. Therefore, pressure sensors that can adjust the sensing range and possess high sensitivity at the same time are highly desirable.

In this paper, we realize piezoresistive pressure sensors with tunable sensing ranges by self-patterned-spacer design. The vertical carbon nanotube (CNT) arrays are grown by a plasma-enhanced chemical vapor deposition (PECVD) method and the two CNT-array layers embedded in PDMS are separated by the patterned spacer. The pressure sensors with large initial resistance designed can change the response thresholds from 300 Pa to 6.56 kPa while keeping the high sensitivity simultaneously. These properties are realized by adjusting the thickness of the spacer and the length of the CNTs. Due to the large specific surface area of the vertical CNT arrays, the resistance of the pressure sensors changes dramatically within a very narrow pressure range, which can achieve a high sensitivity of about 50 kPa^−1^. The proposed pressure sensors also exhibit rapid response/relaxation time of 24/32 ms, and good long-term stability with durability test over 10 000 loading/unloading cycles, while the realized pressure sensors with small initial resistance designed show a typical piezoresistive characteristic. For applications, the pressure sensors with large initial resistance designed are suitable for a flexible keyboard with anti-mis-touch function and flexible data acquisition systems with shielding-noise functions, respectively. On the other hand, the pressure sensors with small initial resistance designed not only show the capability of monitoring mechanical forces such as pressing, bending and torsion, but also can be attached to the skin to monitor human physiological signals such as clenching, arm bending, walking, running, drinking, coughing and speaking. Moreover, the specialty and advantage of this self-patterned-spacer design is that the silicon wafer not only serves as a substrate for CNT growth, but also acts as a natural mask for the patterning of the spacer without affecting the original CNT arrays. This self-patterned spacer separation method is also generally applicable to the other existing works to enhance or adjust their sensor performance, showing great flexibility. The sensing-range-tunable pressure sensors realized by self-patterned-spacer design work as a prototype and show great potentials in flexible wearable electronics such as health monitoring, human–machine interaction and the IoTs.

## Experimental

### Growth of vertical CNT arrays

The metallic multi-walled CNT arrays were synthesized by a PECVD method. First, we used thermal growth to form 1 μm silicon dioxide on a clean silicon wafer. Then, 2 nm Fe as a catalyst was deposited on the Si/SiO_2_ substrate. A radio frequency (RF) PECVD apparatus (Kejing OTF-1200X-II-80SL) was used for CNT growth. After the chamber was evacuated to high vacuum, the sample in the chamber was heated to 700 °C with 7 sccm argon (Ar) and 3 sccm hydrogen (H_2_). After that, methane (CH_4_) was injected into the chamber at a flow rate of 15 sccm and with the RF power fixed at 250 W. During this process, CNTs precipitated from catalyst particles following tip growth theory. After the growth, the chamber was cooled down to room temperature with 7 sccm Ar. The length of vertical CNTs can be controlled by adjusting the growing time.

### Pressure sensor fabrication

**Fig. 1:**
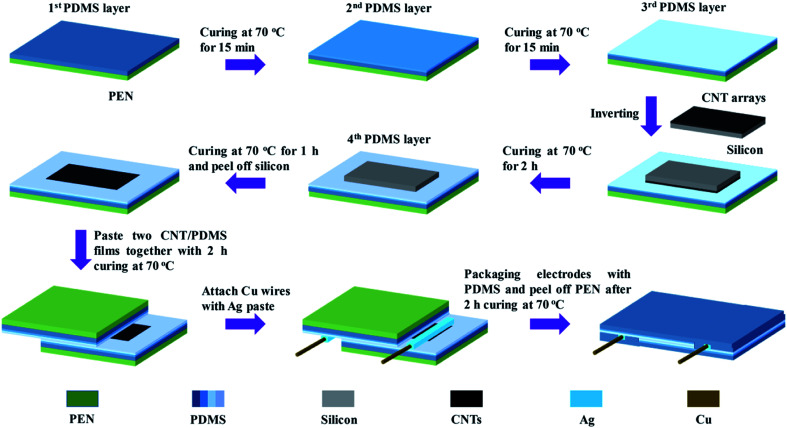
Fabrication process of the pressure sensor realized by CNT/PDMS films and self-patterned-spacer design.

Fig. 1 depicts the fabrication process of the pressure sensors realized by the vertical CNT arrays embedded in PDMS and self-patterned-spacer design. The stable structure of CNT arrays embedded in PDMS was achieved by transfer process with three layers of PDMS.^28^ Before fabrication, the PDMS (Dow Corning Sylgard 184) liquid was obtained by fully stirring the mixture of the base and curing agent in a 10 : 1 mass ratio, followed by the degassing process in a vacuum chamber for 20 min. Subsequently, the PDMS layer of about 400 μm thickness was spin-coated onto a polyethylene naphthalate (PEN) substrate. Then, it was put into an oven at 70 °C for 15 min to achieve a semi-cured state and connected with the second PDMS layer of about 100 μm thickness, which helped to improve the flatness of the substrate. Similarly, after 15 min curing at 70 °C, the third PDMS layer with a thickness of about 10 μm was spin-coated onto the second layer to work as the transfer medium. After that, the prepared CNT array sample with an area of about 1.5 cm × 3 cm was immediately inverted and gently placed into uncured PDMS. During the baking process in an oven 70 °C for 2 h, the neat CNT arrays would partially sink into the third PDMS layer. At the same time, the liquid PDMS would be adsorbed into the dense CNT arrays under the influence of the capillary wetting effect. Since then, the stable structure of CNT arrays embedded in the PDMS film was formed.

As the spacer used for adjusting the initial contact state between the upper and bottom CNT layers, the fourth PDMS layer with a designed thickness was spin-coated on the place without CNT arrays. It is especially designed in this process that the silicon wafer, which is the substrate for CNT growth, plays the role of a natural mask to form a patterned spacer. Since the interfacial bonding between PDMS and CNT arrays is much stronger than that between the Si/SiO_2_ substrate and CNT arrays, the silicon substrate can be peeled off easily from the flexible CNT/PDMS film after 1 h of curing. During the fabrication process of pressure sensors, the edges of two prepared CNT/PDMS films were cut off, and then the prepared CNT/PDMS films were interlaced and sealed together, forming a solid structure after baking for 2 h. The copper wires were connected to the CNT arrays exposed at both sides of the sensor with silver waste. After curing, the electrodes were encapsulated by the PDMS liquid to enhance the connection of electrodes and the films. Finally, the two PEN films were peeled off and the pressure sensors were completed. The spacer fabrication method is called self-patterned-spacer design.

### Characterization and measurement

The morphologies and structures of the vertical CNT arrays and CNT/PDMS films were characterized using a field emission scanning electron microscope (FE-SEM, ZEISS SUPRA®55). The Raman spectra were measured using a Raman spectrometer (Horiba Labram HR Evolution). To test the mechanical and electrical performance of the pressure sensors, the static and dynamic pressures were applied using a manually actuated pressure testing machine (ZHIQU Precision Instruments ZQ-21A) and an electrodynamic force tester (ZHIQU Precision Instruments ZQ-990B), respectively. During the testing, the current changes in the pressure sensors were measured using a semiconductor characterization system (Keithley 4200-SCS) and a high-speed digital multimeter (Keithley DMM6500) using instrument-control software (Keithley KickStart-2.2.1). It should be stated that the maximum measurable resistance of the test equipment is 100 MΩ.

## Results and discussion

### Microstructure characterization

**Fig. 2:**
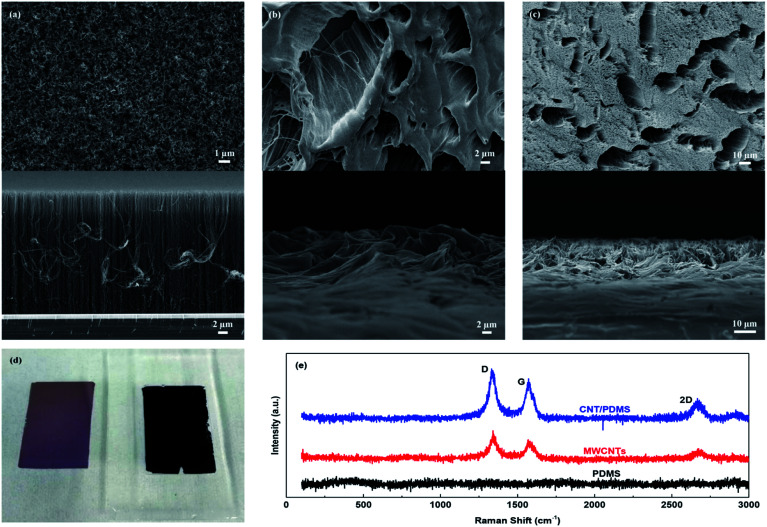
Characterization of the vertical CNT arrays and CNT/PDMS films. Top and cross-sectional SEM views of (a) 30 μm vertical CNT arrays, transferred CNT/PDMS films with a CNT length of (b) 30 μm and (c) 40 μm, respectively. (d) Photograph of the transferred CNT/PDMS film and the silicon substrate after being peeled off. (e) Raman spectra of the vertical CNT arrays, pure PDMS, and the transferred CNT/PDMS film.

The characterization of the vertical CNT arrays and CNT/PDMS films can be seen from SEM images. Fig. 2a shows the top and cross-sectional SEM views of 30 μm CNT arrays grown by the PECVD method. From the clean surface, the grown CNTs show few impurities and good alignment. The CNT diameter is inversely proportional to the catalyst thickness. Since the thickness of the Fe catalyst is only 2 nm, the diameter of CNTs is generally less than 20 nm, resulting in a high density. The CNT arrays are interconnected by a large number of conductive paths, which contribute to a high electroconductivity. The top and cross-sectional SEM views of the transferred CNT/PDMS films with 30 μm and 40 μm CNT arrays are shown in Fig. 2b and c, respectively. Due to the capillary wetting effect, PDMS are adsorbed into CNT arrays, forming a variety of microstructures. The morphologies of these microstructures are mainly determined by the penetration extent of PDMS into CNT arrays. When longer CNTs are used, infiltrated PDMS will not affect the CNT shape on the surface, that is, the surface morphology of the film will not be much changed. At the same time, the contact joints of CNT arrays are not damaged, so the CNT/PDMS film can keep the good conductivity of CNTs, which also determines the piezoresistive characteristics of the sensors. As shown in Fig. 2d, the CNT arrays are almost entirely transferred to PDMS, demonstrating a high transfer efficiency. The Raman spectra of the vertical CNT arrays, pure PDMS and the transferred CNT/PDMS film are shown in Fig. 2e. The Raman spectra of CNTs include a D band at 1342 cm^−1^, a G band at 1580 cm^−1^, and a 2D band at 2691 cm^−1^, which represent the amorphous carbon, the graphitized carbon and the stacking order of the nanosheets, respectively,^29,30^ while pure PDMS has no obvious peak in the Raman shift ranging from 100 to 3000 cm^−1^. All the bands of CNTs can be found in the Raman spectra of the transferred CNT/PDMS film, indicating that there are exposed CNT tips on the surface of the film. Comparing the Raman spectra of CNTs and the CNT/PDMS film, the intensity ratio of the G band and D band increases after transfer. This is because the root of the as-grown CNTs with better graphitization was transferred to the surface of the film.

### Performance of the pressure sensors

**Fig. 3:**
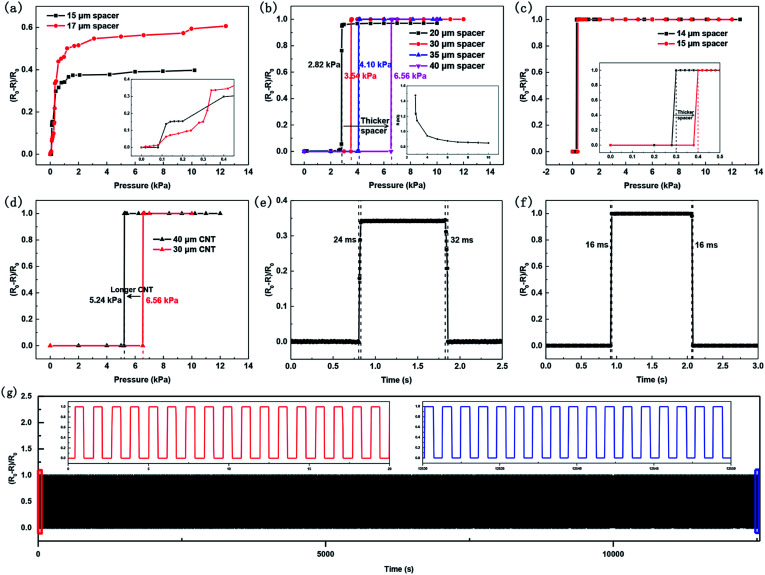
Performance of the pressure sensors realized by self-patterned-spacer design and the vertical CNT arrays embedded in PDMS. Relative changes in resistance *versus* pressure for the sensors using CNTs with different lengths or spacers with different thicknesses. (a) Sensors with 30 μm-length CNTs, and 15 and 17 μm-thick spacers, respectively. (b) Sensors with 30 μm-length CNTs, and 20, 30, 35 and 40 μm-thick spacers, respectively. The inset shows the resistance change of the sensor with a 20 μm-thick spacer as the pressure increases after the response threshold. (c) Sensors with 20 μm-length CNTs, and 14 and 15 μm-thick spacers, respectively. (d) Sensors with a 40 μm-thick spacer, and 30 and 40 μm-length CNTs, respectively. The response/relaxation time for the pressure sensors with (e) smaller and (f) larger initial resistances designed. (g) Long-term stability test of the pressure sensor under loading/unloading cycles of over 10 000 times.

For the characterization of the sensor sensitivity, the relative change in resistance is defined as (*R*_0_ − *R*)/*R*_0_, where *R*_0_ and *R* are the resistances of the pressure sensor without and with applied pressures, respectively. Thus, the sensitivity was calculated as (*R*_0_ − *R*)/*R*_0_/Δ*P*, where Δ*P* is the pressure applied to the sensors. As mentioned above, the performance of pressure sensors can be adjusted by changing the thickness of spacers, and their working mechanisms can be simply divided into two categories. When the thickness of the spacer is relatively thin, the upper and bottom CNT layers are in contact with each other at the beginning, resulting in a small initial resistance of the pressure sensors. As shown in Fig. 3a, these sensors have characteristic curves of typical piezoresistive pressure sensors, and the sensitivity decreases as the pressure increases. As comparison, the pressure sensor with a 17 μm spacer shows a sensitivity of 0.86 kPa^−1^ within the range of 0–0.4 kPa, higher than that of the one with a 15 μm spacer. This is because the thicker the spacer is, the fewer the initial contacts between the upper and bottom CNT layers are, and thus, the larger the initial resistance of the pressure sensor is. This causes a relatively large change in resistance when applying the same pressure, and thus, the sensitivity of the pressure sensor will be higher. When the thickness of the spacer is continuingly increased, the upper and bottom CNT layers are separated from each other at the beginning, and the initial resistance of the pressure sensors is very large (∼100 MΩ). Only when a certain pressure is reached, the upper and bottom CNT layers will touch each other and the sensor will reach a small resistance (∼kΩ). That is, the resistance of the sensor will change greatly within a very narrow pressure range, resulting in a very high sensitivity. Fig. 3b demonstrates that the response thresholds of these pressure sensors can be adjusted from 2.82 to 6.56 kPa by changing the thickness of the spacers with a sensitivity close to 50 kPa^−1^, which is the maximum measurable limit of the test equipment we used. The inset shows the resistance change of the pressure sensor as the pressure increases after the response threshold, which is similar to the pressure sensor with small initial resistance designed. Fig. 3c shows that the minimum response thresholds of the pressure sensors can reach 300 Pa. Similarly, the response thresholds of the pressure sensors can also be controlled by varying the length of the CNT arrays, as shown in Fig. 3d. In theory, as long as the suitable spacers are chosen, we can adjust the response thresholds to any wanted pressure value. As shown in Fig. 3e and f, the proposed pressure sensors also show a fast response/relaxation time, which are 24/32 ms and 16/16 ms for pressure sensors with smaller and larger initial resistance designed, respectively. In general, due to the considerable viscoelasticity of flexible materials, the relaxation time of many pressure sensors is relatively long.^25,31^ The realized pressure sensors can overcome this disadvantage by designing the spacer and the air layer in a confined space. Obviously, after the pressure is removed, the arched upper CNT/PDMS film will be restored quickly by the elastic force from this unique structure. Fig. 3g shows the representative durability test of the sensors under variable pressures. The performance of the pressure sensor shows no significant regression under loading/unloading cycles of over 10 000 times, demonstrating its good long-term stability. Compared to pressure sensors, whose active materials and flexible materials are mixed together or two different layers fabricated by coating, spin-coating, sputtering and soaking,^32–36^ the robust CNTs in this work are embedded in PDMS and the upper and bottom CNT/PDMS films are separated by a spacer and an air layer in a confined space, leading to an advantageous combination of CNTs and PDMS while avoiding excessive squeezing. This design demonstrates good long-term stability of the CNTs and the pressure sensors simultaneously.

### Applications of the pressure sensors

**Fig. 4:**
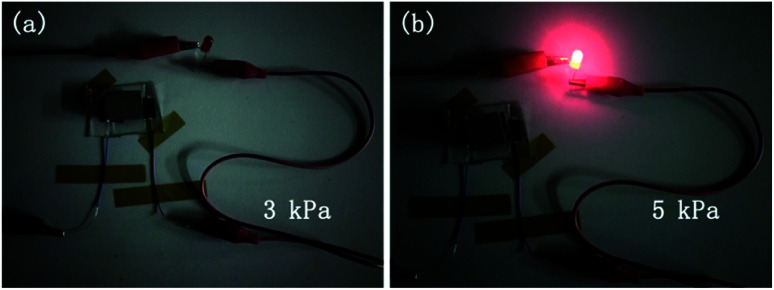
Switching characteristics of the pressure sensor with large initial resistance designed. The LED bulb responds differently when the sensor is applied with pressures of (a) 3 kPa and (b) 5 kPa, respectively.

**Fig. 5:**
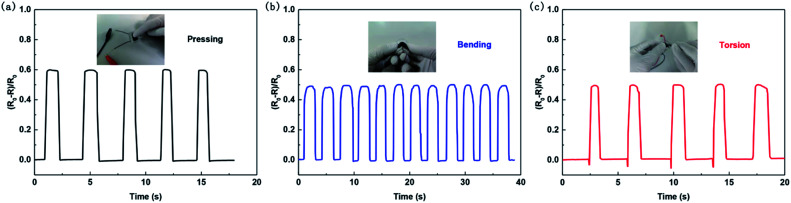
Mechanical force-sensing ability of the pressure sensor with small initial resistance designed. Relative changes in resistance *versus* dynamic loading and unloading cycles of (a) pressing, (b) bending and (c) torsion.

In practical applications, pressure sensors are expected to be sensitive enough and repeatable for both subtle and large pressures, so it is valuable for the pressure sensors to possess a response-range-adjustable design, so that they can fit for the various application needs while still keeping the high sensitivity simultaneously. For the pressure sensors with large initial resistance designed, the upper and bottom CNT layers are separated from each other at the beginning, and the sensor is almost in an insulated state. The pressure sensor will not start working until a certain pressure is reached. To further visualize the switching characteristic of this pressure sensor, we built a complete circuit including a constant voltage source (Agilent E3620A), an LED bulb, and a pressure sensor applied with different pressures of 3 kPa (Fig. 4a) and 5 kPa (Fig. 4b), respectively. It can be found that when the applied pressure is 3 kPa, the LED bulb is at off state, since the upper and bottom CNT layers cannot contact each other, and the resistance of the sensor is extremely large. When the applied pressure is 5 kPa, the LED bulb emits bright light, since the upper and bottom CNT layers contact and the circuit is working. The response of the LED bulb under different pressures proves that the sensor will only start working when the applied pressure reaches its critical switching point.

The switching characteristic of the pressure sensors with large initial resistance designed makes them applicable in many advanced applications. We take the flexible keyboard as an example for future integration of flexible systems. At present, the response thresholds of most flexible keyboards based on pressure sensors are very small, which subjects to mis-touch and outputs wrong information when hands move.^37,38^ Here, the realized pressure sensors with large initial resistance designed will only be activated when the pressures reach the pressure thresholds of the sensors, thus avoiding the mis-touches and noises. At the same time, the pressure sensors have good long-term stability, which are more suitable for strong-durability applications like flexible keyboards. Besides, in flexible IoT applications, massive data will be generated, transported, and stored. However, a lot of useless data will be collected and a large amount of energy will be consumed under the interference of noise. The realized pressure sensor shows a strong filtering effect due to its pressure threshold. When used together with other flexible data acquisition systems, the sensors can filter unnecessary noises, reduce the amount and difficulty of data processing from the hardware level, and avoid unnecessary power consumption. What is more, the pressure sensors can act as alarm devices, especially for the health monitoring of the elderly or children.

The pressure sensors with small initial resistance designed have a different working mechanism from those with large initial resistance designed. As is observed, the former with a typical piezoresistive characteristic are suitable for health monitoring applications. The results in Fig. 5 show that the pressure sensor with small initial resistance designed can detect dynamic pressing (Fig. 5a), bending (Fig. 5b) and torsion (Fig. 5c), demonstrating its ability to detect external forces.

**Fig. 6:**
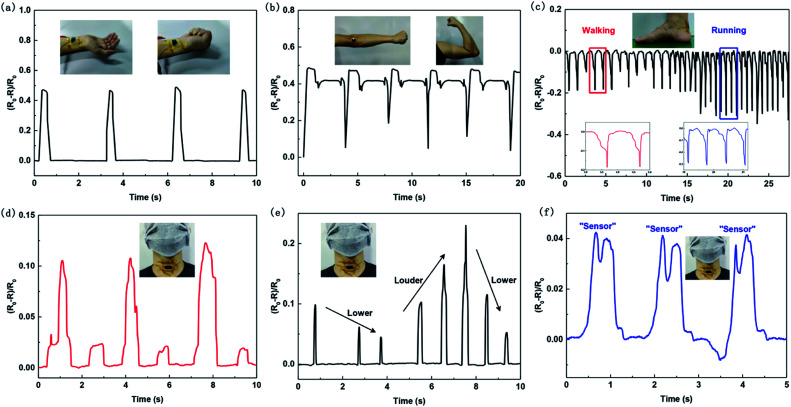
Monitoring of human conditions by the pressure sensors with small initial resistance designed. Relative changes in resistance response of the pressure sensors *versus* (a) fist clenching, (b) arm bending, (c) foot motions, (d) drinking, (e) coughing and (f) speaking.

Furthermore, the pressure sensors with small initial resistance designed can be attached to the human body to detect the human motions. Fig. 6a shows the response of the pressure sensor *versus* fist clenching motion, showing high signal-to-noise ratio. Fig. 6b shows the response of the pressure sensor attached to the arm joint to detect arm bending. The result is a differentiable curve with some small peaks derived from arm joints. Fig. 6c shows the response of the pressure sensor when attached to a volunteer's heel to monitor foot motions. The result shows a trend in resistance changing direction different from other detections in Fig. 6. When the pressure sensor was tightly attached to a person's uneven heel, the sensor was initially in a compressed state. When walking or running, the feet would be lifted, the compression state of the sensor would be relieved, and thus the resistance of the sensor would increase. Comparing the curves of walking and running, the resistance change of running is bigger than walking, and the frequency of running is obviously faster. We also stuck the sensor onto the throat to detect the information during drinking, coughing, and speaking. As Fig. 6d demonstrates, each motion of drinking causes a relative resistance change of about 0.1 for the pressure sensor. At the same time, a small peak appears in the middle of each drink. This is because person's throat has a slight adjustment after drinking. In the coughing detection shown in Fig. 6e, the volunteer coughed at low and high frequencies alternately, with a change in intensity at the same time. When the frequency of coughing is low, the time interval between peak and peak is increased. When the intensity of coughing is increased, not only does the amplitude of the curve become larger, but also the time required for coughing increases. All the detailed information about coughing can be detected by the sensor, demonstrating its potential to distinguish different coughing habits. When the volunteer repeatedly spoke words of “sensor”, similar patterns were clearly recorded by the pressure sensor, as shown in Fig. 6f. The pattern contains split peaks matched with the disyllable pronunciation of “sensor”. Evidently, the pressure sensor is good at vocal detections for both motions and pronunciations, showing the potential for applications in artificial throat, health monitoring and voice recognition.

## Conclusions

In conclusion, we have proposed and realized sensing-range-tunable piezoresistive pressure sensors by self-patterned-spacer design. The vertical CNT arrays are grown by a PECVD method and the two CNT layers embedded in PDMS are separated by the self-patterned spacer in the novel design. The pressure sensors with large initial resistance designed can change the response thresholds from 300 Pa to 6.56 kPa while maintaining a high sensitivity, by controlling the thickness of the spacer and the length of CNTs. Due to the large specific surface area of the vertical CNT arrays, the resistance of the pressure sensors changes dramatically within a very narrow pressure range, achieving a high sensitivity of about 50 kPa^−1^. Benefiting from the designs of the self-patterned spacer and the advantageous combination of CNTs and PDMS, the pressure sensors also exhibit a rapid response/relaxation time of 24/32 ms and good long-term stability with durability test over 10 000 loading/unloading cycles. While the realized pressure sensors with small initial resistance designed show a typical piezoresistive characteristic. Moreover, the self-patterned design and fabrication method of the spacers are simple and controllable, showing potentials to be applied in the existing works to further enhance or adjust the performance of those pressure sensors. With this design, many piezoresistive pressure sensors with high sensitivity in low pressures can completely shift their performance to certain higher-pressure ranges to meet various applications, showing great flexibility. For some existing microstructured pressure sensors, this method can be applied to fabricate spacers before peeling off the mold to adjust the device performance without affecting the original microstructures using the mold, which plays the role of a natural mask like the silicon wafer in this work. In practical applications, the pressure sensors with large initial resistance designed are suitable for the anti-noise applications that require pressure thresholds such as flexible keyboards to avoid mis-touch and flexible data acquisition systems to filter unnecessary noise and save power consumption, respectively. However, the pressure sensors with small initial resistance designed not only show the capability of detecting mechanical forces, but also can be attached to the skin to monitor human physiological signals. As a prototype, the sensing-range-tunable pressure sensors show great potentials in various flexible wearable electronics such as health monitoring, human–machine interaction, the IoTs, and beyond.

## Conflicts of interest

There are no conflicts of interest to declare.

## Supplementary Material
